# Where to restore: Connectivity forest for spatial prioritization in forest landscape restoration

**DOI:** 10.1016/j.isci.2025.113263

**Published:** 2025-08-08

**Authors:** Xiaoming Wang, Johan Svensson, Bengt Gunnar Jonsson, Navinder J. Singh, Jakub W. Bubnicki, Andrés Lopéz-Peinado, Per Angelstam, Grzegorz Mikusiński, Jonas Ardö

**Affiliations:** 1 Department of Wildlife, Fish and Environmental Studies, Swedish University of Agricultural Sciences (SLU), 901 83 Umeå, Sweden; 2 Department of Natural Sciences, Design and Sustainable Development, Mid Sweden University, 851 70 Sundsvall, Sweden; 3 Population Ecology, Mammal Research Institute, Polish Academy of Sciences, 17-230 Białowieża, Poland; 4 Department of Forestry and Wildlife Management, University of Inland Norway, Campus Evenstad, 2480 Koppang, Norway; 5 School for Forest Management, Swedish University of Agricultural Sciences (SLU), 739 21 Skinnskatteberg, Sweden; 6 Grimsö Wildlife Research Station, Swedish University of Agricultural Sciences (SLU), 730 91 Riddarhyttan, Sweden; 7 Department of Physical Geography and Ecosystem Science, Lund University, 223 62 Lund, Sweden

**Keywords:** environmental science, environmental management, ecology

## Abstract

Forest loss, fragmentation, and transformation negatively impact forest biodiversity and ecosystem functionality worldwide. Improving landscape intactness and connectivity through restoration is critical. Determining where to restore remains, however, a challenge. As an approach for prioritizing restoration areas, we define connectivity forest (CFs) as forests outside recognized high conservation value forests (HCVFs) with capacity to support landscape-scale connectivity and green infrastructure (GI) functionality. Across a 1.3 million-ha watershed in boreal Sweden, we identified approximately 130,500 ha of CFs, equal to double the current HCVF area. By integrating CFs with consecutively lower HCVF probabilities, we demonstrate planning implementation at lower to higher ambition levels and identified specific restoration hotspots to guide local-scale restoration planning. Our CF approach has clear implications for efficient spatial targeting of restoration in forest regions where improving conservation in balance with continued forestry for wood production is required to meet national and international biodiversity and environmental goals.

## Highlights

• Connectivity forest (CF) informs landscape- and patch-scale restoration planning
• A total of 130,500 ha of CFs was mapped across a 1.3 million-ha watershed in boreal Sweden
• Restoration hotspots within CFs identify focal areas for local restoration efforts
• Integrating CFs effectively strengthens connectivity of the conservation network

## Graphical abstract

**Figure:**
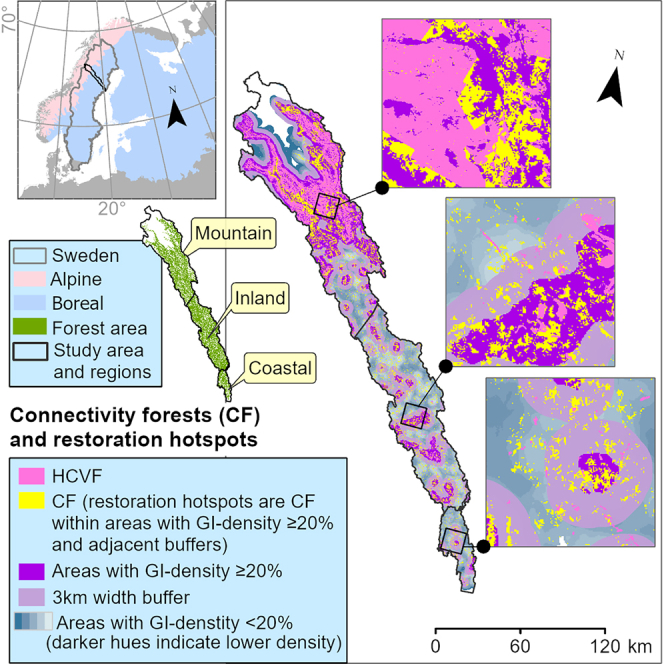

## Introduction

Forest loss, fragmentation, and transformation impose critical challenges globally,^1^^,^^2^^,^^3^ leading to significant deficiencies in biodiversity and ecosystem functionality.^4^^,^^5^ Changes in strategic, tactical, and operational approaches to forest governance and management are thus urgently needed,^6^^,^^7^^,^^8^ as highlighted in high-level initiatives such as the recently ratified EU Nature Restoration Regulation^9^ and Biodiversity Strategy,^10^ the Kunming-Montreal Global Biodiversity Framework,^11^ and the UN Decade on Ecosystem Restoration.^12^ Forests with high or potentially high conservation values have precedence as conservation and restoration target areas to maintain or improve their conservation status and meet agreed objectives.^7^^,^^13^ The spatial allocation of these target areas is crucial to enhance the overall conservation status of forest landscapes.^14^^,^^15^^,^^16^^,^^17^ However, determining where to restore remains a key strategic, tactical, and operational planning challenge.^18^ Forest and environment authorities, landowners, land managers, and policymakers need to base decision making on evidence-based information. In regions with long-term and extensive industrial use of forests, such as in Sweden, remnant stands of high conservation value are few and fragmented, necessitating spatial restoration planning that involves forests with different levels of anthropogenic footprint.^16^ Restoration efforts must be carefully balanced by considering both conservation objectives and continued forestry for wood production.

Restoration is a core component in functional green infrastructure (GI).^19^^,^^20^ In GI planning, large and intact forest areas serve as nodes contributing to resilient ecosystems and functional connectivity,^21^^,^^22^^,^^23^ natural pools of ecosystem services, and climate change adaptive capacity.^24^^,^^25^ The concept of intact forest landscapes^4^ emphasizes their importance. Strategically planned and spatially explicit restoration can strengthen these nodes and support linkages across the landscape.^20^^,^^26^

The boreal forest is the second largest terrestrial biome, comprising nearly one-third of the global forest area.^27^ It supports rich biodiversity, diverse and critical ecosystem services, and the highest terrestrial living biomass globally.^27^^,^^28^^,^^29^^,^^30^ However, vast natural and semi-natural boreal forest landscapes have undergone extensive transformation into simplified wood biomass cropping systems, resulting in severe habitat fragmentation, biodiversity loss, and degradation of indigenous and other sociocultural values.^27^^,^^31^^,^^32^

Sweden harbors a large share of the European continent and European Union forests, approximately 12% and 18%, respectively, corresponding to 28 million ha.^33^ About 20 million ha are forests perceived suitable for forestry, with the remaining area being protected, not available, or on sites with too low wood production capacity.^34^ Despite increased attention to environmental considerations since the 1990s,^35^ the prevailing forestry model focusing on a rotation system optimizing wood biomass yield has resulted in critical environmental, conservation, and restoration challenges. The remaining contiguous areas of intact boreal forest landscapes are primarily located in the hinterlands^36^ of the Scandinavian Mountains Green Belt.^37^^,^^38^ Agreed national, European, and international forest biodiversity and environmental goals are not met.^16^^,^^39^ The current formal protection share, 9% of the total forest area, is not sufficient and geographically imbalanced, with 58% located in the mountain foothills and subalpine forests in Sweden, whereas only 3%–5% is found elsewhere.^40^ Thus, there is an evident and comprehensive need for both active and passive restoration actions^41^ to achieve functional GI at both landscape and patch scales.^3^^,^^42^ The Swedish Environmental Protection Agency estimated that an additional 4 million ha of forests need restoration and/or protection to align with the agreed EU Biodiversity Strategy.^43^

The recently published high conservation value forests (HCVFs) national-scale prediction model^44^ provides innovative opportunities to address how much and where to locate restoration and additional protection. The model’s training data were a national HCVF database^45^ covering all forest areas in Sweden with confirmed high conservation value, regardless of protection or not. This model predicts the probability (i.e., “relative likelihood”^44^) ranging from 0.0 to 1.0 of any 1-ha area with ≥50% forest cover being HCVFs. Unlike many previous approaches to map large land surfaces (e.g., continental-scale) using coarse spatial resolution^4^^,^^46^ or only local areas with specific conservation interests,^47^ or to employ a binary classification of conservation status (e.g., high vs. non-conservation value), this model is particularly well suited for GI planning of forest landscapes. It enables the expansion of connectivity from existing HCVF patches into surrounding forests with 1-ha resolution information on HCVF likelihood.^44^

Leveraging the “actionable” nature of the model output, we introduce the concept of “connectivity forests (CFs)” as an approach for spatial identification, delineation, and prioritization of potential restoration areas. We define CFs as forest areas with high to intermediate likelihood of harboring HCVF qualities, yet not recognized in the national HCVF dataset, as the dataset is not comprehensive across the Swedish forest landscape. When actively or passively restored, CFs have the potential to expand or connect confirmed patches in the HCVF dataset, thereby increasing the density of the conservation network and supporting GI. By progressively incorporating CF areas with lower probabilities of being HCVFs, we outline pathways that accommodate increasing restoration and conservation ambitions, demonstrating changes in both the area and density of the resulting GI network. Simultaneously targeting restoration, conservation, and continued forestry for wood production enables addressing the balance between these competing objectives.

**Figure 1:**
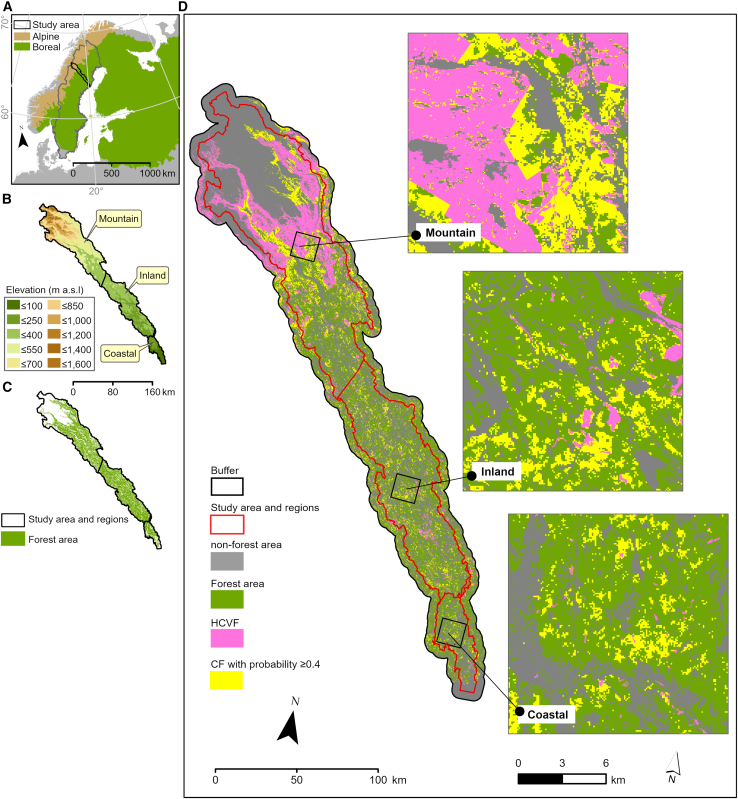
Study area and regions, HCVF baseline, and CFs (A) Location of the study area: Vindelälven watershed, northern Sweden. (B and C) (B) Elevation (m a.s.l) and (C) forest area according to National Land Cover Database^49^ across the three study regions (Mountain, Inland, Coastal). The division between the Coastal and Inland regions is defined by the northwestern border of the two County of Västerbotten municipalities adjacent to the Gulf of Bothnia (i.e., Umeå and Vännäs) to Vindeln and Lycksele municipalities, and between the Inland and Mountain regions, by the border of the northwestern Sorsele municipality. The Mountain region also includes a part of Arjeplog municipality, County of Norrbotten. (D) Distribution of HCVF baseline and CFs with HCVF model^44^ generated probability ≥0.4 in the study area with a 5-km buffer. The three insert squares show selected representative areas within each region.

As a case study, we used the 1.3 million-ha Vindelälven River watershed in northern Sweden, stretching 450 km from northwest to southeast across two ecoregions: the subalpine region of the Scandinavian Mountain Range and the boreal region extending to the Gulf of Bothnia (Figure 1). Across this gradient of mountain foothills, inland, and coastal regions, we (1) mapped the regional and forest-type distribution of CF areas, (2) assessed successive expansion of the conservation network by gradually incorporating CF areas with progressively lower probabilities of being HCVF into the existing HCVF, (3) demonstrated improved landscape connectivity and habitat functionality, and (4) identified numerous restoration hotspots within CF areas, cumulatively covering extensive forest areas, thereby linking a landscape perspective to local-scale restoration planning and practice. Given the varying forest landscape histories, past and present forestry footprints, conservation status, and forest ownership in the study area,^48^ our study provides innovative insights on “where to restore,” particularly for balancing restoration and conservation ambitions in forest landscapes where wood biomass–oriented forestry is likely to continue. As northern Sweden’s situation mirrors that of many other forest regions, our approach holds promise for widespread adoption in the much-needed, targeted, and well-balanced forest and landscape restoration planning.

## Results

### Methods summary

We divided the study area into three regions: Mountain, Inland, and Coastal (Figure 1), reflecting gradients in biogeographical variation, human impact, and forestland ownership, i.e., a dominance of public land in the mountains, private forest company land in the inland, and non-industrial private land in the coastal region^50^ (see Methods S1 for a detailed introduction of the study area).

We mapped CFs as forest areas with a minimum HCVF probability of 0.4 outside HCVF areas in the national database^45^ (hereafter HCVF baseline) across the three regions and four forest types: spruce, pine, deciduous, and all forests combined (Figure 1). This 0.4 threshold corresponds to the previously reported minimum value for formally and voluntarily protected forests.^51^ We divided CFs into six classes with 0.1 intervals and stepwise inserted these into the HCVF baseline. We then assessed the changes in area and forest type composition of the expanded HCVF baseline.

We visualized changes in the GI density,^52^^,^^53^ a simple yet indicative metric of landscape-scale structural connectivity, also used in the Swedish national GI-oriented landscape analysis.^54^ GI density was chosen over other more comprehensive metrics, such as cumulative current density,^15^^,^^55^ also because it aligns well with the study’s focus on demonstrating a CF-based planning approach rather than providing an in-depth assessment of connectivity dynamics. GI density calculated the percentages of cumulative area of the HCVF baseline and stepwise inserted CF classes relative to the study area, filtered by moving windows with radii of 3 and 1 km, respectively. We compared the GI density value distribution and the increase in density medians across study regions and forest types using the 3-km results.

Applying a 20% lower threshold for habitat functionality,^16^^,^^56^^,^^57^ we delineated areas with GI density ≥20% to assess changes in functional habitats. Finally, we identified restoration hotspots among the mapped CF areas. Inserting these hotspots onto the HCVF baseline adds areas with GI density ≥20%, thereby improving habitat functionality.

### Regional differences in area distribution and forest type composition

The absolute CF area of all four forest types was highest in the Mountain region, lower in the Inland region, and lowest in the Coastal region, except for pine-dominated CF classes, which were ≥0.5 and ≥0.4 in the Inland region (Table 1). When weighted by the total area of each forest type, the proportional CF area followed the same regional pattern. Although lower than in the Mountain region, CF classes ≥0.4 in the Inland and Coastal regions still accounted for 12% and 11% of their respective total forest areas (for all forest). For specific forest types, these proportions increased up to 22% and 21% (for pine forest).

**Table 1:** Area of nested Connectivity Forest classes (CF-class; HCVF model–generated probability ≥0.9 to ≥0.4) classes across regions and forest types, in hectares (ha) and proportions (%) of the total area of the corresponding forest type and study region (Mountain, Inland, Coastal), and area increase (%) on top of the HCVF baseline area of all forest in the corresponding study region

Forest type	Nested CF class	Area (ha)			Area (%)			Area increase (%)		
		Mountain	Inland	Coastal	Mountain	Inland	Coastal	Mountain	Inland	Coastal
All forest	CF ≥ 0.9	9,318	42	–a	3	0^b^	–	7	0	–
	CF ≥ 0.8	20,840	834	8	6	0	0	16	8	1
	CF ≥ 0.7	31,298	2,962	106	9	1	0	25	29	7
	CF ≥ 0.6	49,074	9,314	744	14	3	1	39	93	47
	CF ≥ 0.5	63,851	19,704	2,256	18	6	4	50	196	142
	CF ≥ 0.4	80,981	42,901	6,562	23	12	11	64	426	412
Spruce	CF ≥ 0.9	4,652	22	–	2	0	–	4	0	–
	CF ≥ 0.8	9,797	237	3	4	0	0	8	2	0
	CF ≥ 0.7	13,409	804	27	7	1	0	11	8	2
	CF ≥ 0.6	17,849	2,363	168	11	3	2	14	23	11
	CF ≥ 0.5	21,710	4,763	512	16	7	5	17	47	32
	CF ≥ 0.4	26,865	9,502	1,259	25	16	15	21	94	79
Pine	CF ≥ 0.9	1,074	15	–	5	0	–	1	0	–
	CF ≥ 0.8	2,761	483	3	11	1	0	2	5	0
	CF ≥ 0.7	4,371	1,753	62	16	2	0	3	17	4
	CF ≥ 0.6	7,313	5,365	436	21	5	3	6	53	27
	CF ≥ 0.5	10,792	11,274	1,237	25	11	9	8	112	78
	CF ≥ 0.4	16,576	24,700	3,449	31	22	21	13	245	217
Deciduous	CF ≥ 0.9	3,576	5	–	2	0	–	3	0	–
	CF ≥ 0.8	8,112	93	–	5	0	–	6	1	–
	CF ≥ 0.7	13,134	384	14	9	1	0	10	4	1
	CF ≥ 0.6	23,127	1,286	124	15	2	1	18	13	8
	CF ≥ 0.5	30,128	2,876	440	20	5	3	24	29	28
	CF ≥ 0.4	35,507	6,720	1,596	23	12	10	28	67	100

a Dash (−) shows no area.

b Zero (0) shows any area below 0.5 ha.

In total, close to 81,000 ha of CF area was mapped in the Mountain region, with deciduous forest, i.e., the mountain birch alpine tree line forest, contributing the largest share. In the Inland and Coastal regions, the CF area was close to 43,000 ha and 6,500 ha, respectively, with pine forest contributing the largest share in both regions. Additionally, the CF class ≥0.9 for any forest type, and the CF class ≥0.8 for deciduous forest, did not occur at all in the Coastal region.

### Greater area expansion in Inland and Coastal regions, particularly of pine forest

CF insertion resulted in substantial area increases relative to the HCVF baseline in all study regions and for all forest types. In particular, the increases in the Inland and Coastal regions could potentially exceed 400%, with CF of pine forest, alone, contributing over 200% (Table 1). When comparing across the three regions after inserting all CF classes ≥0.7, the proportional area increases in the Inland and Coastal regions were consistently higher than in the Mountain region for all four forest types, with the highest increases generally occurring in the Inland region. Among the three specific forest types, only the area ratio of pine forest consistently increased with each stepwise CF insertion across all three study regions (Figure S1), with the most evident change observed in the Coastal region with a ratio increase from 0.20 to 0.48.

### Pine as the primary potential contributor to GI density increases in the Inland and Coastal regions

**Figure 2:**
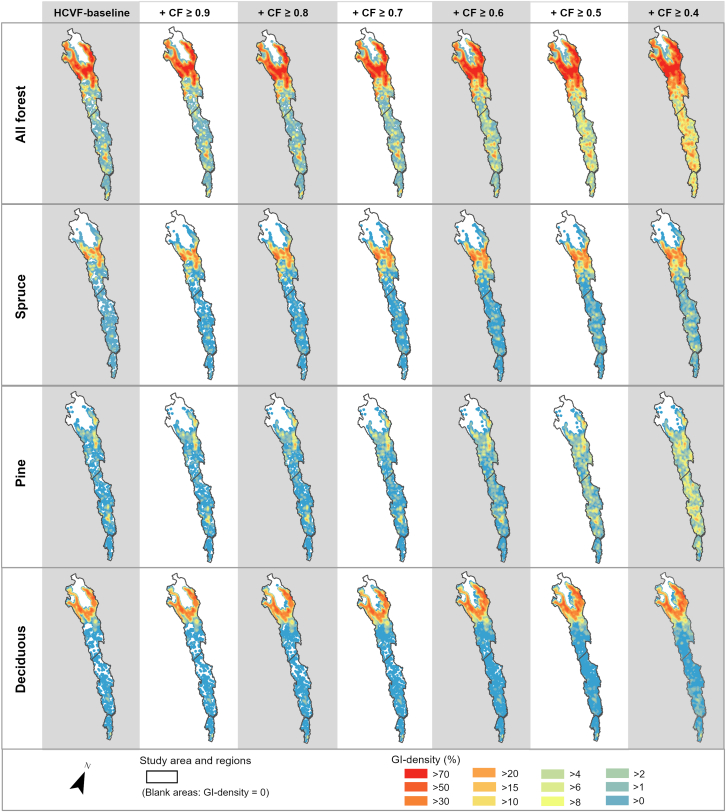
Changes in green infrastructure (GI) density from HCVF baseline through stepwise insertion of nested CF classes for all forest and spruce, pine, and deciduous forests GI density is based on a circular moving window with a 3-km radius. See Figure S2 for results using a 1-km radius.

**Figure 3:**
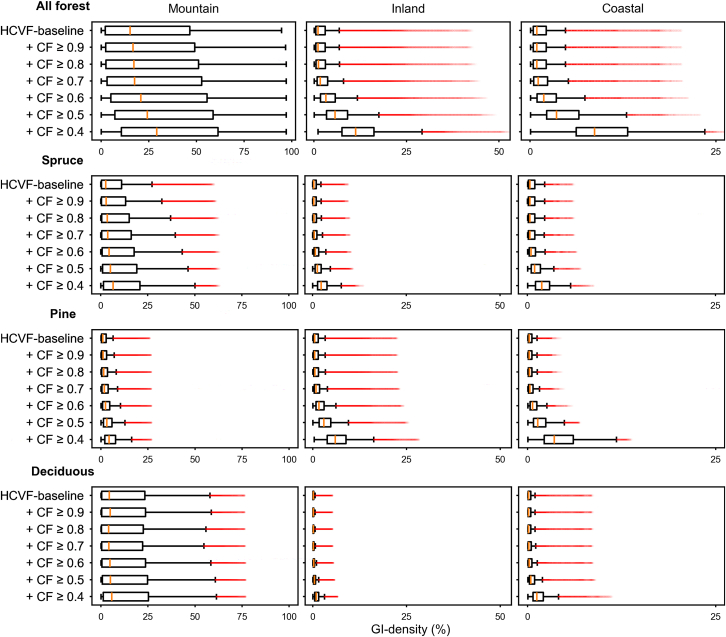
Changes in GI density from HCVF baseline through stepwise insertion of nested CF classes for all forest and spruce, pine, and deciduous forests in the Mountain, Inland, and Coastal regions Each boxplot displays the minimum, median, and first and third quartiles, with whiskers extending to within 1.5 times the interquartile range and outliers shown in red. The GI density is based on a circular moving window with a 3-km radius. Note that the x axis scale varies between the regions.

**Figure 4:**
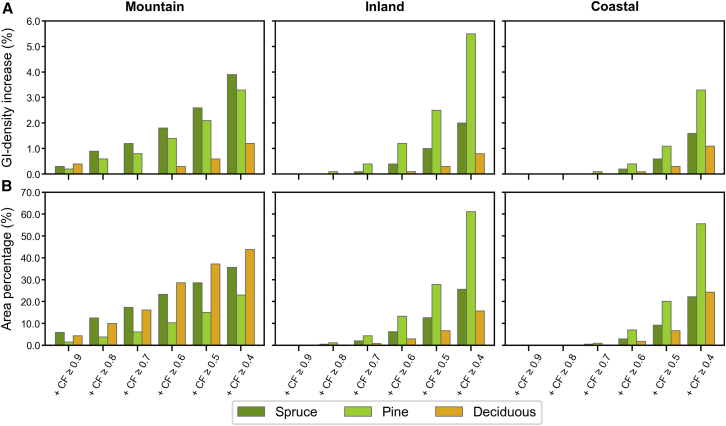
Median increases (%) in GI density (A) and cumulative area (%) of inserted CFs (B) from HCVF baseline through insertion of nested CF classes for spruce, pine, and deciduous forests in the Mountain, Inland, and Coastal regions Increases in density medians are calculated relative to the medians of the HCVF baseline for the corresponding forest type and region. Area percentages (%) are calculated relative to the total CF area of all forest within the corresponding study region (specified in Table S1). GI density is filtered by a circular moving window of 3-km radius. See Table S1 for bar values.

The GI density gradually increased from the HCVF baseline through the insertion of CF classes across the study area, as illustrated in Figure 2 (see also Figure S2). For all forest, inserting CF classes ≥0.6 closed the most evident gaps in GI density (i.e., areas with a GI density = 0) in the Coastal and Inland regions as well as in the lower part of the Mountain region. Inserting CF classes ≥0.6 also closed the density gaps for pine forests throughout the study area, but not for spruce and deciduous forests. By comparing the distribution patterns of density values within each study region, we found that the increase in GI density was primarily contributed by spruce forest in the Mountain region, and by pine forest in both the Inland and Coastal regions (Figures 3 and 4; see also Table S1). In the Inland region, pine forest showed the highest median density increase at CF ≥ 0.4 (Figure 4). However, a higher increase in GI density did not consistently result from a proportionally larger CF area inserted. As shown in the Mountain region, the density increase associated with deciduous forest was much lower than that of spruce and pine forests, despite the largest allocation of CF area input to deciduous forest.

**Figure 5:**
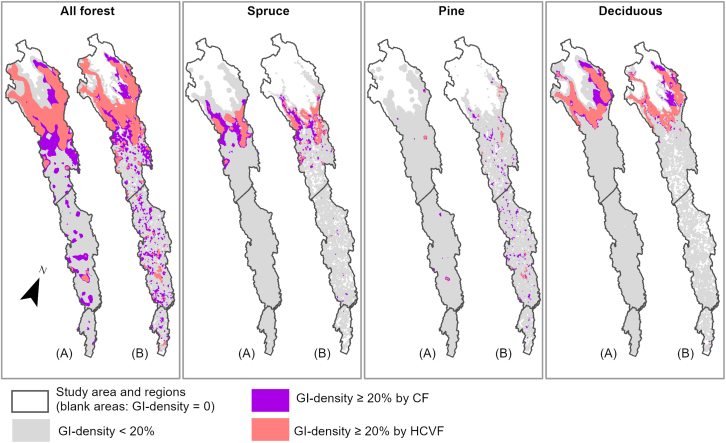
Area of all forest and spruce, pine, and deciduous forests with GI density ≥20% following the insertion of all CFs with HCVF model probability ≥0.4 The density is filtered with a circular moving window with a 3-km (A) or 1-km radius (B). Areas illustrated in light red indicate patches with GI density ≥20% in the HCVF baseline, and those in violet, additional patches with GI density ≥20% as a result of the CF insertions. See Figure S3 for maps following each CF insertion. See Table S2 for areas with GI density ≥20%.

Based on the HCVF baseline alone, areas with GI density ≥20% already accounted for 26% and 41% of the total area with GI density >0, filtered by the 3- and 1-km moving windows, respectively (Figures 5 and S3; Table S2). CF insertions increased these area proportions to 40% and 43%, concentrated in the Mountain region and maintained primarily by spruce and deciduous forests. Across the entire study area, patches with a GI density ≥20% were almost exclusively added by pine forests. These patches were much fewer in number, smaller in size, and more geographically isolated compared with those in the Mountain region.

### Numerous restoration hotspots and substantial hotspot areas identified

**Figure 6:**
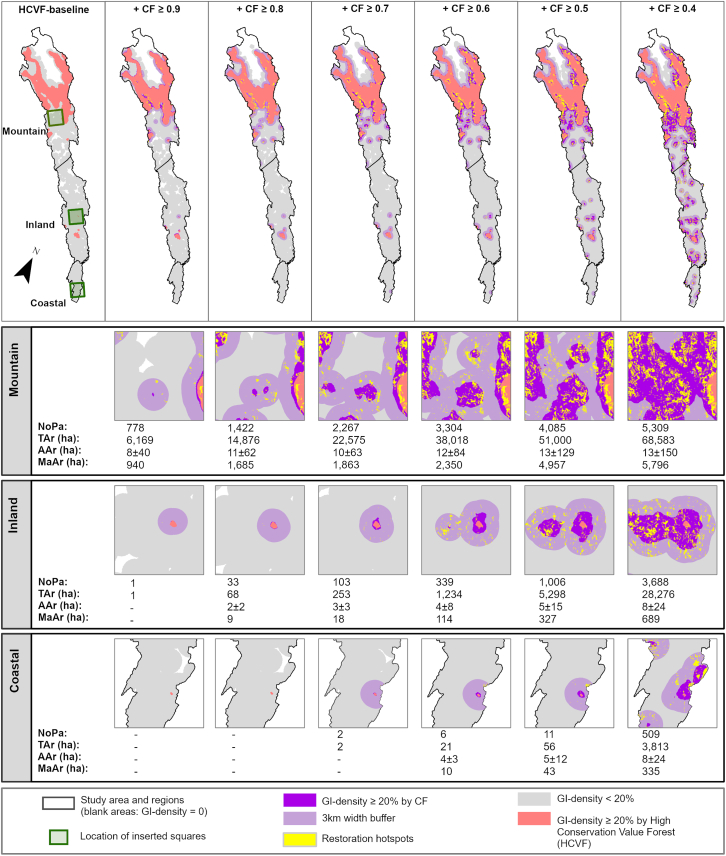
Restoration hotspots (yellow), identified as CF patches in areas with a GI density ≥20% (dark violet) and their surrounding 3-km width buffer (light violet) throughout the stepwise CF insertions, for all forest in the Mountain, Inland, and Coastal regions of the study area The GI density is filtered by a circular moving window with a 3-km radius. The restoration hotspots are illustrated at a higher spatial resolution in the three squares (see also Figure 1). The statistics summarize the number of patches (NoPa), total area (TAr; ha), average area with standard deviation (AAr; ha), and maximum patch area (MaAr; ha) of all the restoration hotspots in each region. A patch denotes a contiguous cluster of pixels, where contiguity is valid between any focal pixel and any of its eight surrounding pixels.

A total of 9,506 restoration hotspots (≥1 ha), covering 100,672 ha, were identified across the study area, in proximity to areas already displaying a GI density ≥20% before any CF insertions. The number, total area, average area, and largest patch area of these hotspots decreased from the Mountain to the Inland and Coastal regions (Figure 6). Nevertheless, 3,688 and 509 restoration hotspots covering 28,276 and 3,813 ha were identified in the Inland and Coastal regions, respectively, with the largest patch areas being 689 and 335 ha.

## Discussion

### Connectivity forest approach to identify restoration hotspots

Focusing on “where to restore,” we find the CF approach applicable for prioritizing restoration areas at various spatial scales. In addition to the HCVF baseline, our study identifies an additional 81,000, 43,000, and 6,600 ha of CF areas in the Mountain, Inland, and Coastal regions, respectively. This showcases a significant restoration potential for strategic and operational GI planning. The stepwise nesting of CF areas with successive lower HCVF probabilities provides a foundation for adaptive planning that responds to actual restoration needs, ambitions, and implementation premises. Thereby, our approach supports restoration planning as an integral part of comprehensive land-use planning^18^^,^^58^ while addressing the central role of forest biodiversity conservation.^59^ It facilitates strategic land-sharing/-sparing implementations to effectively manage the diverse values and functionalities of forests.^60^ Additionally, as the HCVF baseline is not comprehensive across the Swedish forest landscape,^45^ the CF approach aids in the *in situ* identification and validation of previously unknown or undocumented HCVF areas. Thus, the CF approach maps forest patches suitable for both active and passive restoration, with the latter indicating set-aside conservation.

We highlight the large restoration potential harbored in CF areas within the Inland and Coastal regions. These CF areas accumulated to over 10% of the total forest areas in these regions, typically dominated by pine forests and with HCVF probabilities below 0.8 (Table 1). Given the much less HCVF baseline areas in these regions compared with the Mountain region (Table S3), further conservation in the Inland and Coastal regions is critical for improving connectivity on the watershed scale.

Among the CF areas, our approach identifies over 5,000, 3,000 and 500 restoration hotspots covering about 68,500, 28,200, and 3,800 ha of forest patches in the Mountain, Inland, and Coastal regions, respectively (Figure 6). These restoration hotspots, when restored, add areas with a GI density of at least 20%, indicating habitat functionality.^16^^,^^56^^,^^57^ Prioritizing these restoration hotspots is therefore likely to connect local-scale restoration initiatives with landscape-scale habitat functionality benefits. Thus, we demonstrate how the CF approach can potentially achieve restoration benefits on multiple spatial scales and assist strategic GI planning. A previous validation^61^ demonstrated that the restoration areas delineated by our CF approach spatially align with “HCVF tracts” identified by the County Administration Board using field data.^54^ Hence, we argue that our CF approach can effectively support landscape restoration and conservation planning in Sweden.

### Restoration challenges and opportunities

Given the high uncertainty associated with actual conservation outcomes of restoration,^62^ restoration actions must be informed, carefully planned, and evaluated. In Sweden, with extensive forest areas, but also facing critical environmental and conservation challenges,^16^^,^^48^^,^^63^ implementation of restoration as a component of sustainable forest management also needs to balance multiple forest values, such as continued wood biomass production, recreation, and traditional and indigenous cultures.^37^

The HCVF dataset indicates that about 18% (Table S3) of the total forest area across the watershed is under conservation attention, whether strictly protected or not. According to recent estimates (Å. Granberg, personal communication), just over 5% (40,000 ha) of the total forest area (757,104 ha) is under strict protection. These values fall short of the committed quantitative goals, such as the 20% target in the EU Nature Restoration Regulation.^9^ The current level of protection is clearly insufficient for ensuring functional connectivity among habitat and forest patches^16^^,^^37^^,^^53^^,^^64^^,^^65^ and fails to emphasize key biodiversity aspects^59^ in representative forest habitat types.^66^ Our study identifies CF areas corresponding to 17% of the total forest area, which indicates substantial opportunity for restoration and further protection. Most of the CF areas are found in the Mountain region. This is both an indication of and a result of the intact forest landscapes protected along the Scandinavian Mountains Green Belt.^16^^,^^37^^,^^38^ Consequently, the need for prioritizing restoration efforts in the Mountain region is less urgent. However, the situation changes already in the mountain foothills southeast transition zone^38^ where conditions are similar to the Inland region. The CF areas identified here, as well as those in the Inland and Coastal regions, provide critical starting points for reinforcing GI across the entire watershed.

Given the limited area of known HCVF in the Inland and Coastal regions, our CF approach indicates a substantial increase in area on top of the HCVF baseline in these two regions, potentially expanding their respective HCVF baseline areas by over 400% (for all forest, see Table 1). Indeed, in severely fragmented landscapes, even protecting smaller patches provides conservation benefits and supports the provision of important ecosystem services.^23^^,^^37^^,^^67^^,^^68^^,^^69^ If restoration is to proceed, however, planning will have to be balanced within the local socioeconomic context, including ongoing forestry,^18^^,^^70^ which remains a key challenge. The identified CF areas, especially those with lower probabilities approaching to 0.4, are unlikely to be set aside from production-oriented forestry at large scales and will likely continue to be under harvesting pressure. Therefore, assuming that active restoration measures allow limited harvesting and wood sales to facilitate economic aspects, our results, consistent with numerous studies,^18^^,^^60^^,^^71^^,^^72^ advocate for diversified forest management over passive area preservation as a feasible way to promoting restoration. Furthermore, the high precision in mapping CFs enables the identification of individual ownership properties and landowners, facilitating inquiries into their interest and willingness to participate.

### Restoration value and potential of pine forest in achieving watershed-scale connectivity

Some forest types are largely ignored in conservation.^16^^,^^73^ This appears to be the case with pine forests in our study area. In contrast to their low contribution to the HCVF baseline, our results reveal the potential for pine forest restoration at the watershed scale, particularly in the Inland and Coastal regions. Since pine is the dominant tree species, pine forests potentially offer a more feasible restoration pathway compared with spruce, and even more clearly with deciduous forests. Besides harboring biodiversity values, pine forests serve as core areas for traditional cultures in boreal regions.^74^^,^^75^ Older, more open pine-dominated forests support a rich lichen flora, which serves as critical winter-grazing resources for traditional Sami reindeer husbandry.^76^

While advocating for increased attention to the restoration potentials and values of pine forests, we acknowledge the need for restoration of deciduous and spruce forests also. Historically, industrial forest production has altered the distribution, quantity, and structure of the entire forest landscape,^77^ with systematic clear-cutting followed by monoculture plantations of pine and spruce, as well as systematic mechanical and chemical removal of deciduous trees, being the most prevailing factors.^78^ Consequently, the proportion of natural and near-natural spruce and deciduous forests is lower than in their natural state,^79^ with the networks of spruce and deciduous forest habitats continuously shrinking.^80^ The scarcity of habitats associated with deciduous species is considered the most pressing GI challenge.^15^

### Implementing localized restoration hotspots to assist strategic restoration planning

Although broad-scale restoration planning frameworks are essential, restoration efforts typically focus on local areas tens to hundreds of hectares in size.^81^ Therefore, strategically prioritized and well-informed localization of restoration actions is crucial for maximizing conservation efficiency and gains.^81^

Our CF approach explicitly localized potential restoration hotspots that contribute to landscape-scale GI density increase and connectivity benefits and thus addressed the challenge of prioritizing restoration sites to achieve overarching goals.^82^ Local-scale restoration efforts and landscape restoration effects are interlinked. For other CF patches with the same or higher HCVF probability, but not identified as restoration hotspots, restoration would rather be based on their local, intrinsic characteristics than on their landscape contribution. Thereby, the CF approach allows informed prioritization based on spatially identified actual conservation needs and premises.

From the Mountain to the Coastal region, the identified restoration hotspots gradually decrease in size. In the Inland and Coastal regions, these hotspots are generally smaller, with an average patch size of less than 10 ha and the largest patches covering 350–700 ha. In the boreal forest landscape of northern Sweden, as in many forest regions worldwide, only fragments of natural and near-natural forests remain.^22^^,^^48^^,^^53^^,^^83^ Under these circumstances, conservation planning and restoration of representative, functional, and well-managed forests for biodiversity protection will rely on small forest areas.^84^ Although small areas are also important in biodiversity conservation,^23^^,^^68^^,^^84^^,^^85^^,^^86^ their ecological functionality benefits from being connected in a functional forest network.^14^^,^^17^ The gradient explored in this study clearly shows the importance of integrating small area protection and restoration in the regional conservation scheme.

### Conclusion

The spatially explicit information we provide at the forest-type level can support efforts to directly meet or build the capacity to meet the principles of forest and forest landscape restoration.^87^ It also complies with the adopted EU Nature Restoration Regulation and other high-level restoration ambitions, specifically regarding connectivity aspects. Although an HCVF model like the one we utilized may not be available in all countries and regions, our CF approach illustrates the potential and feasibility of systematically, quantitatively, and explicitly mapping forests with restoration capacity. Hence, our study underscores a conceptual opportunity and workflow adaptable to diverse datasets, landscape knowledge, and GI-planning needs. As a planning basis, field inventory and further implementation can be directed to specific landscape segments, increasing the precision, accuracy, and transparency while lowering the costs.

### Limitations of the study

Our identification of CFs and restoration hotspots was based on multiple thresholds. We acknowledge that these thresholds may not be applicable across different biodiversity aspects, forest regions, or spatial scales. For instance, we used a GI density threshold of ≥20% to indicate functional networks, a criterion supported in previous studies as a rule of thumb.^56^ Notably, CF areas were defined using a probability threshold of ≥0.4. This may be ecologically reasonable^51^ but likely reflects an overly idealized level of restoration ambition given current political and socio-economic realities. However, although alternative thresholds and buffer sizes could have been chosen, we provide a relevant basis for discussing restoration planning. Given the generic nature of our approach, these thresholds and criteria can easily be adjusted to suit specific circumstances and needs. Additionally, future implementation of this approach should seek integrating more specific ecological attributes of boreal forests or other targeted forest ecosystems, such as species composition. Further refinement should address more specific conservation challenges under the changing climate.

## Resource availability

### Lead contact

Further information and requests for resources should be directed to and will be fulfilled by the lead contact, Johan Svensson (johan.svensson@slu.se).

### Materials availability

This study did not generate new unique materials.

### Data and code availability

• Data reported in this article will be shared by the lead contact upon request.
• The article does not report any new code.
• Any additional information required to reanalyze the data reported in this article is available from the lead contact upon request.

## Acknowledgments

We thank Åsa Granberg from the County Administrative Board of Västerbotten, Sweden, for her support in providing conservation estimates and expert perspectives specific to the study area. X.W., J.S., B.G.J., N.J.S., and A.L.-P. acknowledge the EU Horizon 2020 Research and Innovation Programme SUPERB: Upscaling Forest Restoration - SUPERB (forest-restoration.eu); Grant Agreement number 101036849. J.A. acknowledges the internal funding provided by 10.13039/501100003252Lund University to support this study.

## Author contributions

X.W. designed the study and analyses, conducted the analyses, and wrote and edited the manuscript. J.S. conceptualized the study, designed the analyses; wrote, reviewed, and edited the manuscript; and coordinated the co-work of the author panels and other experts. B.G.J. and N.J.S. contributed to the study’s conceptualization and design and reviewed and edited the manuscript. J.W.B. provided the editable model prediction map and reviewed the manuscript. A.L.-P, P.A., and G.M. reviewed and edited the manuscript. J.A. reviewed the manuscript.

## Declaration of interests

The authors declare no competing interests.

## STAR★Methods

### Key resources table

**Table:** 

REAGENT or RESOURCE	SOURCE	IDENTIFIER
**Deposited data**		

High Conservation Value Forest Model (“Naturvärdeskarta Skog (NVK skog)” in Swedish)	Download: Swedish National Data Service (“SND Svensk nationell datatjänst”) Interactive user-interface: Google Earth Engine	https://snd.se/sv/catalogue/dataset/2024-49/1 https://bubnicki.users.earthengine.app/view/swedentest
High Conservation Value Forest Dataset (“Skogliga värdekärnor” in Swedish)	Geodata catalog of the Swedish Environmental Protection Agency (“Geodatakatalogen”)	https://geodatakatalogen.naturvardsverket.se/geonetwork/srv/swe/catalog.search#/metadata/69655223-a8f3-475c-bf7f-5c0354cd232b
National Landcover Data (“Nationella marktäckedata (NMD)” in Swedish)	Swedish Environmental Protection Agency (“Naturvårdsverket”)	https://www.naturvardsverket.se/verktyg-och-tjanster/kartor-och-karttjanster/nationella-marktackedata

**Software and algorithms**		

ArcGIS Pro 2.7	ESRI	https://pro.arcgis.com/en/pro-app/latest/get-started/download-arcgis-pro.htm
Python 3.11	Python Software Foundation	https://www.python.org/
Rasterio v1.2.10	MapBox	https://rasterio.readthedocs.io/en/stable/intro.html

### Method details

#### Forest type reclassification

We added forest type data using the National Land Cover Data (NLCD).^49^ Six NLCD forest types occur in our study area (Table S4). We then integrated the two mixed forest types (mixed coniferous and mixed deciduous-coniferous) into spruce, pine, and deciduous types, following an area-reallocation scheme shown in Table S5. Thus, in total four forest types, spruce, pine, deciduous, and all forest combined were applied. The forest type distribution across study areas is presented in Table S3, showing that the Mountain region is dominated by deciduous and spruce forests, while the Inland and Coastal regions are dominated by pine forests.

#### High Conservation Value Forest baseline

Second, we employed the HCVF-dataset^45^ (originally compiled in 2016 and updated in 2019 and 2020), rasterized at a 1-ha resolution, to extract the HCVF-baseline. The HCVF-dataset encompasses formally protected, voluntary protected and unprotected forest patches, generally covering natural forests with native tree species, forest continuity, vertical and horizontal complexity, and generally low levels of anthropogenic influence,^45^ mapped by field surveys during several decades without a predefined sampling scheme.^15^ In our study area, the HCVF-baseline accounted for 18% of the total forest area (Table S3), with the majority located in the Mountain region and dominated by deciduous forest (mainly *Betula* spp.) (Figure 1; Table S3). The HCVF-dataset included areas categorized as non-forest by the NLCD (see Table S4). These non-forest areas (in total 31,072 ha) were excluded from our analysis.

#### Identification of Connectivity Forests

We applied a gradient in the HCVF-model probabilities to extract CF from the forest areas outside the HCVF-baseline across the three study regions and four forest types. In doing so, we overlaid the HCVF-baseline, the HCVF-model, and the NLCD map, to extract HCVF-model probability values at 1-ha resolution using ArcGIS Pro 2.7.^88^

The probability quantiles were calculated using Python 3.11^89^ library Rasterio v1.2.10^90^ in order to determine the probability gradient for CF. By comparing the quantiles (Figure S4; Table S6), we observed that in the Inland and Coastal regions, >75% of the forest areas outside the HCVF-baseline had a probability <0.4, while >50% of the forest within the HCVF-baseline had a probability >0.4, thus in line with previous estimate.^51^ We delineated CF outside the HCVF-baseline using the 0.4 threshold (Figure 1).

We divided CF into six classes with a 0.1 value interval and quantified stepwise nested CF of the six discrete classes (i.e., probability ≥0.9, ≥0.8, ≥0.7, …, ≥0.4) across study regions and forest types. The nested stepwise insertion of CF-classes on top of the HCVF-baseline established six levels of spatially explicit conservation scenarios. Following each insertion, we assessed the area of nested CF-classes across forest types and regions. Further, we evaluated the area increases of the expanded HCVF-baseline resulting from each CF-insertion and examined the corresponding changes in forest type composition.

#### GI-density calculation

The spatial re-configuration of the stepwise insertion of CF-classes was assessed using the GI-density metric. GI-density calculates the area percentage of GI-patches filtered by a moving window, here circular moving windows with radii of 3-km and 1-km, respectively. The choice of these two window sizes aligned with those employed in landscape-scale GI-density analyses by the Swedish EPA.^54^ Before applying the moving window, a buffer zone with a 5-km width, encircling the entire outer boundary of the study area, was created to counteract the spatial shrinkage caused by the moving window scanning.

In calculating the GI-density contributed by the three forest types (Spruce, Pine, Deciduous), the CF-areas of mixed coniferous forest were first re-allocated to Spruce and Pine forests. This re-allocation was necessary because GI-density analysis requires explicit spatial distribution of a forest type, whereas the distribution of spruce and pine in mixed coniferous forests is not specified by the NLCD.^9^ Therefore, the partitioning of the mixed coniferous forest area, specified in Table S5, was not applicable any longer. Consequently, all the CF-areas of mixed coniferous forest were repetitively assigned to the CF-areas of both spruce and pine forests, resulting in minor increases in the areas of nested CF-classes for the forest types Spruce and Pine compared to their corresponding values in Table 1. These adjusted areas were also used to calculate the proportional CF-area input (Table S1; see also “changes in GI-density” below).

Further, the CF-areas of mixed stands, whether coniferous mixed or deciduous-coniferous mixed, were assigned a weight factor of 0.5, following the approach proposed by Mikusiński et al.^15^ The consideration is that habitats maintained by mixed forests might be less effective in providing habitat qualities compared with pure stands.^15^^,^^91^

After this two-fold approach to handling CF-areas of mixed forests, the GI-density was calculated in ArcGIS Pro 2.7^88^ using moving window filtering.

#### Changes in GI-density

After each stepwise insertion of CF-classes, we calculated and compared the value distribution of GI-density and the increase in density medians compared with the HCVF-baseline medians, for the three tree-species specific forest types and study regions, filtered by the 3-km moving window. Additionally, we compared the cumulatively inserted CF-areas corresponding to the median increases to determine whether larger inserted CF-areas led to greater increases in GI-density.

#### Changes in areas with GI-density ≥20%

Areas displaying GI-density ≥20% were delineated across the study area. Expansion of such areas was calculated relative to the total area with GI-density >0. The 20% threshold was based on the assumption that over a given forest landscape, the density of the remaining habitat-patches higher than 20% is a general indication of habitat functionality.^16^^,^^57^^,^^58^ This threshold also aligns with the quantitative conservation goal set by the Swedish government.^80^ Although alternative density thresholds could be used, we note that higher density thresholds would strongly limit operational planning opportunities.

#### Identification of restoration hotspots

We mapped restoration hotspots to translate landscape-scale habitat connectivity and functionality benefits into local restoration areas. Essentially, the combined effect of these restoration hotspots and HCVF would add new habitat patches with GI-density ≥20%. The inclusion of the 3-km buffer was necessary because CF-insertions within this zone could influence the density variation of any focal area. We calculated the total number, accumulated area, average size, and maximum size of these hotspots resulting from the insertion of nested CF-classes in each region.

### Quantification and statistical analysis

Quantitative analyses were conducted using Python and ArcGIS Pro 2.7, and the results were shown in Figures 1, 2, 3, 4, 5, and 6.
