# Supplementary material for: Where to restore: Connectivity forest for spatial prioritization in forest landscape restoration
Source: iScience. 2025 Aug 8;28(9):113263. doi: 10.1016/j.isci.2025.113263 (PMC12414835; doi:10.1016/j.isci.2025.113263)
Supplement: Document S1. Figures S1–S4, Tables S1–S6, and Methods S1 [file mmc1.pdf]

iScience, Volume *28*

Supplemental information

Where to restore: Connectivity forest for spatial prioritization in forest landscape restoration

Xiaoming Wang, Johan Svensson, Bengt Gunnar Jonsson, Navinder J. Singh, Jakub W. Bubnicki, Andrés Lopéz-Peinado, Per Angelstam, Grzegorz Mikusiński, and Jonas Ardö

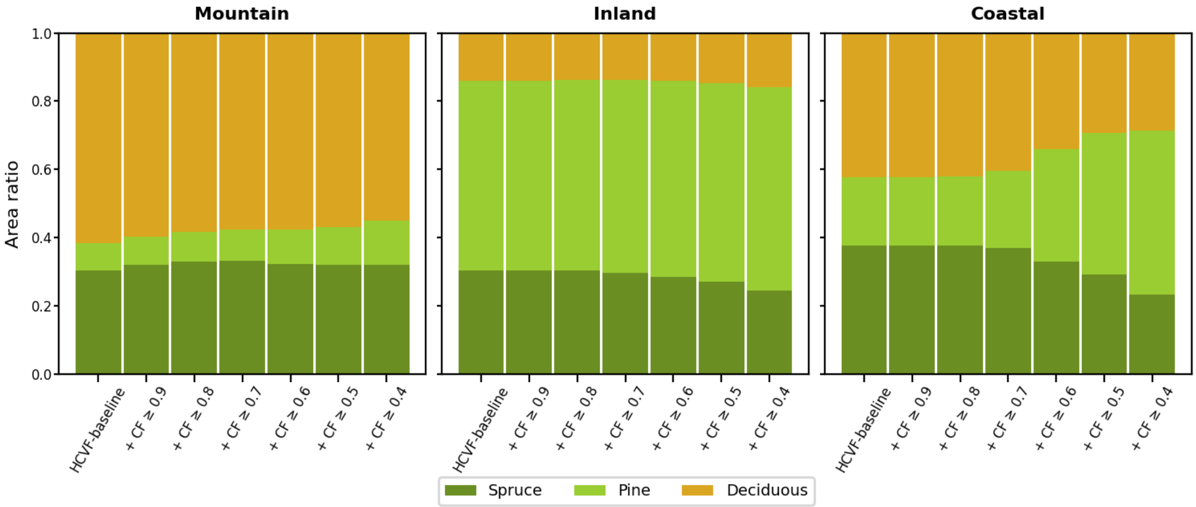

Figure S1. Changes in forest type composition from High Conservation Value Forest (HCVF) baseline through insertion of nested Connectivity Forest (CF) classes in the Mountain, Inland and Coastal region of the study area. Related to Results (subsection “Greater area expansion in Inland and Coastal regions, particularly of pine forest”).

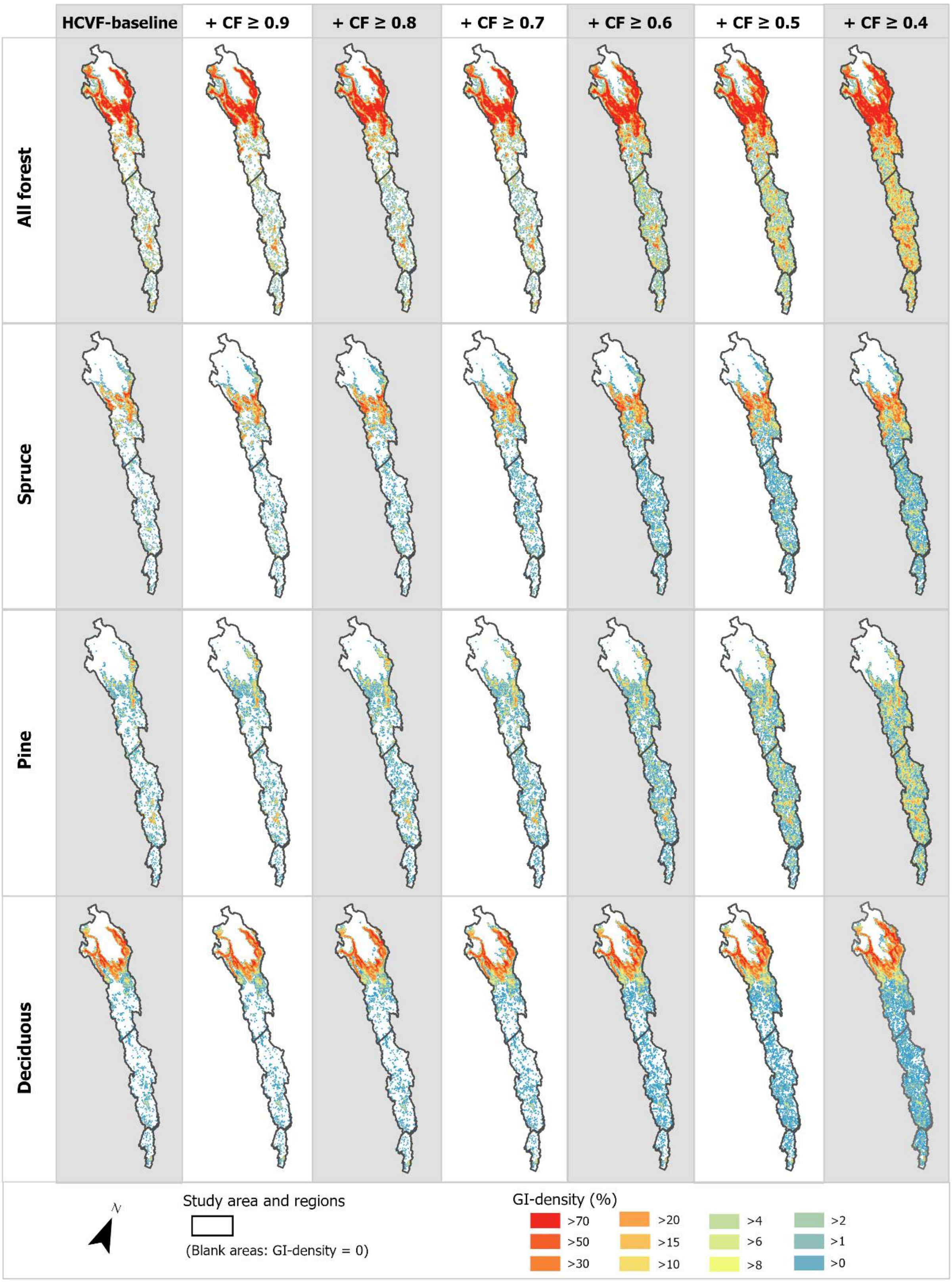

Figure S2. Changes in Green infrastructure (GI) density from High Conservation Value Forest (HCVF) baseline through stepwise insertion of nested Connectivity Forest (CF) classes for all forest, spruce, pine and deciduous forest. GI-density is filtered by a circular moving window with a 1-km radius. Related to Figure 2.

A

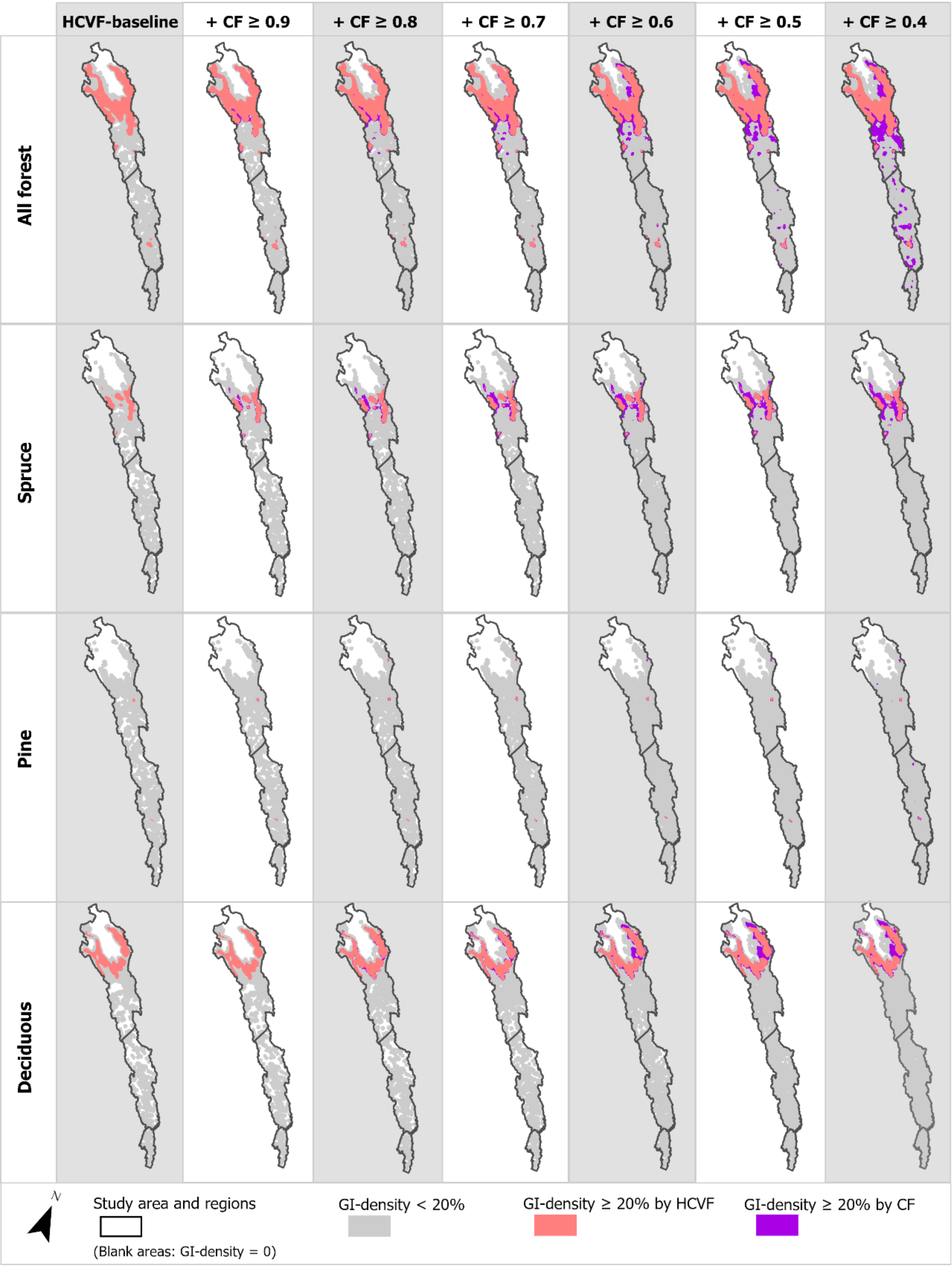

HCVF-baseline
+ CF ≥ 0.9
+ CF ≥ 0.8
+ CF ≥ 0.7
+ CF ≥ 0.6
+ CF ≥ 0.5
+ CF ≥ 0.4
All forest
Spruce
Pine
Deciduous
N
Study area and regions
(Blank areas: GI-density = 0)
GI-density < 20%
GI-density ≥ 20% by HCVF
GI-density ≥ 20% by CF

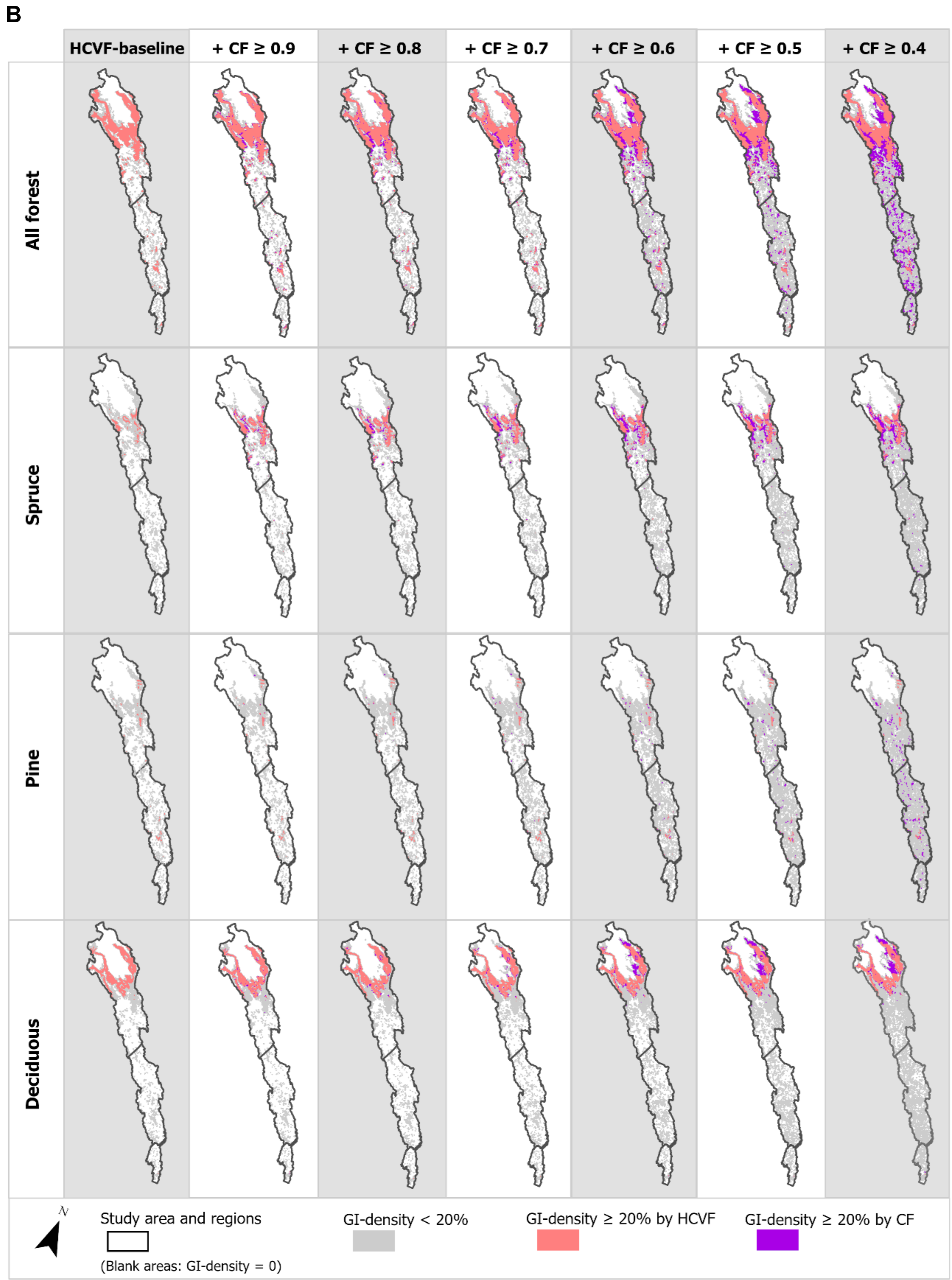

Figure S3. Changes in areas with Green Infrastructure (GI) density ≥20% from High Conservation Value Forest (HCVF) baseline through stepwise insertion of nested Connectivity Forest (CF) classes for all forest, spruce, pine and deciduous forest. GI-density is filtered with a circular moving window with a 3-km (**A**) and 1-km (**B**) radius. Areas illustrated in light red indicate patches with GI-density ≥20% in the HCVF-baseline, in violet indicate additional patches with GI-density ≥20% as a result of the CF-insertions. Related to Figure 5.

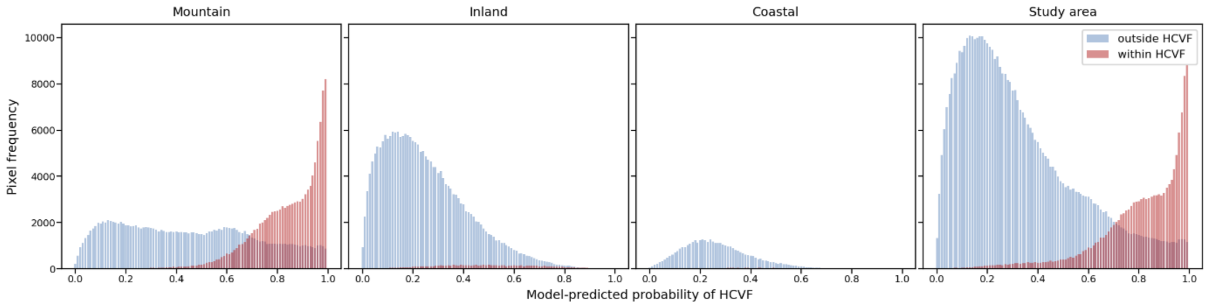

Figure S4. Distribution of High Conservation Value Forests (HCVF) model probability values within and outside HCVF-areas in the three study regions (Mountain, Inland, Coastal) and the entire study area, related to Star Methods (“Identification of Connectivity Forests” under subsection “Method details”).

Table S1. Median increases (%) in Green Infrastructure (GI) density and cumulative Connectivity Forest (CF) area (in % and ha) from High Conservation Value Forest (HCVF) baseline through insertion of nested CF-classes for all forest, spruce, pine and deciduous forest in the Mountain, Inland and Coastal regions of the study area. Related to Figure 4 and Star Methods (subsection “Methods details”).

Forest type	Expanded HCVF-baseline	Mountain			Inland			Coastal		
		median increase (%)	inserted CF-areas		median increase (%)	inserted CF-areas		median increase (%)	inserted CF-areas	
			%	ha		%	ha		%	ha
Spruce	+ CF ≥ 0.9		6	4,809	0	0	24	-[b]	-	-
	+ CF ≥ 0.8	1	13	10,229	0	1	271	0	0	3
	+ CF ≥ 0.7	1	17	14,086	0	2	921	0	1	31
	+ CF ≥ 0.6	2	23	18,914	0	6	2,708	0	3	200
	+ CF ≥ 0.5	3	29	23,146	1	13	5,465	1	9	601
	+ CF ≥ 0.4	4	36	28,914	2	26	11,021	2	22	1,457
Pine	+ CF ≥ 0.9	0	2	1,230	0	0	17	-	-	-
	+ CF ≥ 0.8	1	4	3,192	0	1	517	0	0	4
	+ CF ≥ 0.7	1	6	5,049	0	4	1,870	0	1	66
	+ CF ≥ 0.6	1	10	8,378	1	13	5,710	0	7	469
	+ CF ≥ 0.5	2	15	12,228	3	28	11,976	1	20	1,326
	+ CF ≥ 0.4	3	23	18,625	6	61	26,219	3	56	3,647
Deciduous	+ CF ≥ 0.9	0	4	3,576	0	0	5	-	-	-
	+ CF ≥ 0.8	0	10	8,112	0	0	93	-	-	-
	+ CF ≥ 0.7	0	16	13,134	0	1	384	0	0	14
	+ CF ≥ 0.6	0	29	23,127	0	3	1,286	0	2	124
	+ CF ≥ 0.5	1	37	30,128	0	7	2,876	0	7	440
	+ CF ≥ 0.4	1	44	35,507	1	16	6,720	1	24	1,596

Increases in density medians are calculated relative to the medians of the HCVF-baseline for the corresponding forest type and region. Area percentages (%) are calculated relative to the total CF-area of all forest within the corresponding study region, as specified in Table 1 in the main text. GI-density is filtered by a circular moving window of 3-km radius.

[a] Zero (0) shows any area below 0.5 ha.
[b] Dash (-) shows no area.

Table S2. Changes in areas (%) with a Green Infrastructure density (GI-density) ≥20% from High Conservation Value Forest (HCVF) baseline through insertion of nested Connectivity Forest (CF) classes for all forest, spruce, pine and deciduous forest in the Mountain, Inland and Coastal regions of the study area. Related to Figure 5.

**Forest type**		**Area (%) with GI-density ≥ 20%:**							
		moving window radius = **3 km**				moving window radius = **1 km**			
		Mountain	Inland	Coastal	Study area	Mountain	Inland	Coastal	Study area
All forest	HCVF-baseline	46	2	0[a]	26	57	9	7	41
	+ CF ≥ 0.9	47	2	0	27	57	9	7	41
	+ CF ≥ 0.8	48	2	0	27	57	8	7	40
	+ CF ≥ 0.7	48	2	0	28	56	7	6	37
	+ CF ≥ 0.6	51	2	0	30	56	7	5	36
	+ CF ≥ 0.5	54	4	0	30	58	9	6	37
	+ CF ≥ 0.4	61	14	6	40	61	22	16	43
Spruce	HCVF-baseline	11	-[b]	-	6	17	0.3	0	11
	+ CF ≥ 0.9	13	-	-	7	19	0.3	0	13
	+ CF ≥ 0.8	14	-	-	8	20	0.3	0	13
	+ CF ≥ 0.7	15	-	-	9	20	0.2	0	12
	+ CF ≥ 0.6	16	-	-	9	19	0.2	0	11
	+ CF ≥ 0.5	18	-	-	10	19	0.3	0	11
	+ CF ≥ 0.4	19	-	-	11	20	1	0	11
Pine	HCVF-baseline	0	0	-	0	2	3	-	2
	+ CF ≥ 0.9	0	0	-	0	2	3	-	2
	+ CF ≥ 0.8	0	0	-	0	2	3	-	2
	+ CF ≥ 0.7	0	0	-	0	2	2	-	2
	+ CF ≥ 0.6	0	0	-	0	2	2	-	2
	+ CF ≥ 0.5	0	1	-	0	3	3	0	3
	+ CF ≥ 0.4	1	1	-	1	4	6	2	5
Deciduous	HCVF-baseline	25	-	-	14	31	-	1	21
	+ CF ≥ 0.9	25	-	-	14	31	-	1	21
	+ CF ≥ 0.8	25	-	-	14	30	-	1	19
	+ CF ≥ 0.7	25	-	-	14	28	-	1	18
	+ CF ≥ 0.6	27	-	-	15	28	-	0	17
	+ CF ≥ 0.5	28	-	-	16	28	-	0	16
	+ CF ≥ 0.4	28	-	-	16	28	-	1	15

Area percentages (%) are calculated relative to the total area of GI-density >0 in the corresponding region. GI-density is filtered by a circular moving window with a 1-km and 3-km radius.

[a] Zero (0) shows any area below 0.5 ha.
[b] Dash (-) shows no area.

Table S3. Distribution of forest areas (ha, %) within and outside High Conservation Value Forest (HCVF) across study regions (Mountain, Inland, Coastal) and forest types. Related to Discussion (subsections "Connectivity forest approach to identify restoration hotspots" and "Restoration challenges and opportunities") and Star Methods (subsection "Methods details").

Forest type	**within HCVF:** 138,980 (18%)			**outside HCVF:** 618,124 (82%)			Sum
	Mountain	Inland	Coastal	Mountain	Inland	Coastal	
Spruce	38,803 (5%)	2,949 (<1%)	574 (<1%)	47,280 (6%)	40,831 (5%)	5,415 (1%)	135,851 (17%)
Pine	10,200 (1%)	5,413 (1%)	308 (<1%)	55,977 (7%)	149,424 (20%)	22,243 (3%)	243,564 (32%)
Deciduous	77,829 (10%)	1,352 (<1%)	644 (<1%)	74,623 (10%)	56,175 (7%)	15,547 (2%)	226,170 (30%)
All forest	127,324 (17%)	10,063 (1%)	1,593 (<1%)	228,681 (30%)	333,262 (44%)	56,181 (8%)	757,104 (100%)

Area proportions (%) are calculated relative to the total forest area in the entire study area.

Table S4. Forest types in the study area, classified by the Swedish national landcover database (NLCD). Related to Star Methods (subsection "Methods details").

**NLCD classification of forest types[a] (NLCD code) on forestland [b] in Sweden**	
Pine forest	Pine forest not on wetland (111)
	Pine forest on wetland (121)
Spruce forest	Spruce forest not on wetland (112)
	Spruce forest on wetland (122)
Mixed coniferous	Mixed coniferous not on wetland (113)
	Mixed coniferous on wetland (123)
Deciduous-coniferous mixed	Mixed forest not on wetland (114)
	Mixed forest on wetland (124)
Deciduous forest	Deciduous not on wetland (115)
	Deciduous on wetland (125)
Temporarily not forest [c]	Temporarily non-forest not on wetland (118)
	Temporarily non-forest on wetland (128)

[a] Forestlands are classified into seven major types, based on dominant tree species in combination with the site productivity[1]. A tree species is dominant when its canopy cover is ≥70% of the total canopy cover of any 0.01 ha pixel. Site productivity is divided based on whether a given site supports tree growth of ≥ $1 m^3 ha^{-1} year^{-1}$ over a rotation cycle. The major forest types are further divided by whether they are situated on wet organic soil or mineral soil. Deciduous hardwood forest on wetland (Code 126) and not on wetland (Code 116) do not exist in the study area.

[b] In NLCD, forestland is defined following FAO's standard, i.e., "land spanning more than 0.5 ha with trees higher than 5 meters and a canopy cover of more than 10 percent, or trees able to reach these thresholds in situ. It does not include land that is predominantly under agricultural or urban land use" [2].

[c] Temporarily not forest: open and re-growing clear-felled, storm-felled or burnt areas with trees of < 5m height[1].

Table S5. Re-classified forest types and re-allocated forest areas used in area statistics (Table 1 in the main text and Table S3), based on the Swedish national landcover database (NLCD). Related to Star Methods (subsection “Methods details”).

Foerst type	Area re-allocation	Area (ha)	Sum (ha)
Spruce	All spruce forest (code 112 & 122)	118,661	
	50% of mixed coniferous forest (code 113 & 123)	17,190	135,851
Pine	All pine forest (code 111 & 121)	226,374	
	50% of mixed coniferous forest (code 113 & 123)	17,190	243,564
Deciduous	All deciduous forest (code 115 & 125)	131,918	
	All mixed deciduous-coniferous forest (code 114 & 124)	94,252	226,170
Temp. not forest[a]	All temporarily non-forest (code 118 & 128)		151,520
All forest[b]	Total forest area by NLCD (i.e., all forest above)		757,104

The codes correspond to Table S1.

[a] Temp. not forest: temporarily not forest areas, i.e., open and re-growing clear-felled, storm-felled or burnt areas with trees of < 5m height[1].

[b] All forest: combining forest types of Spruce, Pine, Deciduous and Temp. not forest as one type.

Table S6. Quantiles of the High Conservation Value Forests (HCVF) model probability within and outside HCVF-areas in the three study regions (Mountain, Inland, Coastal) and the entire study area. Related to Star Methods ("Identification of Connectivity Forests" under subsection "Method details").

	Quantile	Mountain	Inland	Coastal	Study area
within HCVF	1st (25%)	0.77	0.33	0.32	0.74
	2nd (50%)	0.88	0.49	0.42	0.86
	3rd (75%)	0.96	0.65	0.53	0.95
	4th (100%)	1.00	0.97	0.88	1.00
outside HCVF	1st (25%)	0.22	0.12	0.17	0.15
	2nd (50%)	0.44	0.22	0.25	0.27
	3rd (75%)	0.67	0.35	0.35	0.46
	4th (100%)	1.00	0.97	0.83	1.00

Methods S1

Supplementary information about study area, related to Results (subsection “Methods summary”).

This study covered the entire Vindelälven watershed, also designated as the UNESCO Vindelälven-Juhttátahkka Biosphere Reserve. Vindelälven is one of four large National Rivers in Sweden, i.e. being protected from hydro-power development. The watershed landscapes are characterized by a mosaic of mountains, forests, waterbodies, and wetlands, and support a rich pool of diverse ecosystem services with local, regional, and global significance, as well as unique natural and cultural values. The indigenous Sami communities, engaged in transhumance reindeer husbandry for millennia, herd reindeer seasonally across this area. Forests and woodlands cover more than half of the total study area, with most forestland of interest for commercial forestry (530,683 ha) and dominated by Scots pine (*Pinus sylvestris*) and Norway spruce (*Picea abies*)[3]. Deciduous forest (mainly *Betula* spp.) is mainly confined to the mountain region.

The forest landscapes have a long history of intensive forest use[4]. Since the establishment of the first Forestry Act in 1903[5], the Swedish forest sector has had a focus on maximized wood biomass yield, and since mid-20th century with systematic clear-cut rotation forestry of even-aged, monoculture stands of mainly Norway spruce and Scots pine[6]. Other tree species, as well as other land uses in forests, have been suppressed[7]. While mountain foothill forests, mainly with Norway spruce, downy birch (*Betula pubescens*) and mountain birch (*Betula pubescens* ssp. *czerepanovii*) forming the alpine tree line, remain largely in a natural or semi-natural state, the inland and coastal forest landscapes are heavily transformed with loss and fragmentation of remaining natural and old forests[8]. These remaining forests of high conservation value are under continued logging pressure[7].

National and regional GI-planning, reflecting EU and international policy ambitions, implies strengthening of existing network of protected areas and further conservation and restoration of forest areas. With the absolute majority of protected area allocated in the northwestern part, including the Vindelfjällen Nature Reserve as the largest reserve in northern Europe (565,000 ha), GI-efforts are particularly challenging in the inland and coastal subregions, where the current share of protection is very low (approx. 5%)[9]. Therefore, with the evident transformation of vast forest areas, ecological restoration is particularly needed in the forest landscapes below the mountain foothills[6].

Supplemental references:

1. Swedish EPA. (2020). National landcover database (NLCD) - Product Description. https://www.naturvardsverket.se/4a018c/contentassets/1db9f81262af4729ac8da0e54fa0808c/nmd2018-product-description-v2-eng.pdf.
2. FAO (2015). FRA 2015 terms and definitions - Forest Resources Assessment Working Paper 180. Food and Agricultural Organization of the United Nations. https://www.fao.org/forest-resources-assessment/past-assessments/fra-2015/en/.
3. Gardeström, J., Grelsson, G., Andersson, J., Norstedt, G., Svensson, J., Nilsson, C., Holmberg, Ö., Sundin, B., Westbergh, S., Myren, A., et al. (2016). Vindelälven-Juhtatdahka biosphere reserve appliation.
4. Jonsson, B.G., Svensson, J., Mikusiński, G., Manton, M., and Angelstam, P. (2019). European Union’s Last Intact Forest Landscapes are at A Value Chain Crossroad between Multiple Use and Intensified Wood Production. https://doi.org/10.3390/f10070564.
5. Nylund, J.-E. (2009). Forestry Legislation in Sweden. The Swedish University of Agricultural Sciences. 14. https://pub.epsilon.slu.se/5503/1/Forestry_legislation_in_Sweden.pdf?origin=publicationDetail.
6. Svensson, J., Mikusiński, G., Bubnicki, J.W., Andersson, J., and Jonsson, B.G. (2023). Boreal Forest Landscape Restoration in the Face of Extensive Forest Fragmentation and Loss. In Boreal Forests in the Face of Climate Change: Sustainable Management, M.M. Girona, H. Morin, S. Gauthier, and Y. Bergeron, eds. (Springer International Publishing), pp. 491-510. https://doi.org/10.1007/978-3-031-15988-6_19.
7. Angelstam, P., Manton, M., Green, M., Jonsson, B.-G., Mikusiński, G., Svensson, J., and Maria Sabatini, F. (2020). Sweden does not meet agreed national and international forest biodiversity targets: A call for adaptive landscape planning. Landscape and Urban Planning 202, 103838. https://doi.org/10.1016/j.landurbplan.2020.103838.
8. Svensson, J., Bubnicki, J.W., Angelstam, P., Mikusiński, G., and Jonsson, B.G. (2022). Spared, shared and lost—routes for maintaining the Scandinavian Mountain foothill intact forest landscapes. Regional Environmental Change *22*, 31. https://doi.org/10.1007/s10113-022-01881-8.
9. Statistics Sweden (2023). Protected nature. https://www.scb.se/en/finding-statistics/statistics-by-subject-area/environment/land-use/protected-nature/.
